# Outcome in Heart Failure with Preserved Ejection Fraction: The Role of Myocardial Structure and Right Ventricular Performance

**DOI:** 10.1371/journal.pone.0134479

**Published:** 2015-07-30

**Authors:** Georg Goliasch, Caroline Zotter-Tufaro, Stefan Aschauer, Franz Duca, Benedikt Koell, Andreas A. Kammerlander, Robin Ristl, Irene M. Lang, Gerald Maurer, Julia Mascherbauer, Diana Bonderman

**Affiliations:** 1 Division of Cardiology, Department of Internal Medicine II, Medical University of Vienna, Vienna, Austria; 2 Section for Medical Statistics, Center for Medical Statistics, Informatics and Intelligent Systems, Medical University of Vienna, Vienna, Austria; University Medical Center Utrecht, NETHERLANDS

## Abstract

**Background:**

Heart failure with preserved ejection fraction (HFpEF) is recognized as a major cause of cardiovascular morbidity and mortality. Thus, a profound understanding of the pathophysiologic changes in HFpEF is needed to identify risk factors and potential treatment targets in this specific patient population. Therefore, we aimed to comprehensively assess the impact of left- and right-ventricular function and hemodynamics on long-term mortality and morbidity in order to improve risk prediction in patients with HFpEF.

**Methods and Results:**

We prospectively included 142 consecutive patients with HFpEF into our observational, non-interventional registry. Echocardiography, cardiac magnetic resonance imaging and invasive hemodynamic assessments including myocardial biopsy were performed at baseline. We detected significant correlations between left ventricular extracellular matrix and left ventricular end-diastolic diameter (r = -0.64;p = 0.03) and stroke volume (r = -0.53;p = 0.04). Hospitalization for heart failure and/or cardiac death was observed over a median follow up of 10 months. The strongest risk factors were reduced right ventricular function (adj. HR 6.62;95%CI 3.12- 14.02;p<0.001), systolic pulmonary arterial pressure (adj. HR per 1-SD 1.55;95%CI 1.15- 2.09;p = 0.004) and the pulmonary artery wedge pressure (adj. HR per 1-SD 1.51;95%CI 1.09–2.08; p = 0.012). The area under the ROC curve for right ventricular function was 0.63, for systolic pulmonary arterial pressure 0.75, and for pulmonary artery wedge pressure 0.68.

**Conclusion:**

The current study emphasizes the importance of right ventricular function and pulmonary pressures on outcome in patients with HFpEF providing pathophysiological insights into the hemodynamic changes in HFpEF.

## Introduction

Heart failure with preserved ejection fraction (HFpEF) has been established as a major cause of cardiovascular morbidity and mortality. While identification of risk factors and advances in treatment led to a significant reduction in mortality during the last decades in patients with heart failure with reduced ejection fraction, the mortality in patients with HFpEF remained virtually unchanged.[1, 2] Thus, a profound understanding of the pathophysiologic changes in patients with HFpEF is crucially needed to identify risk factors and potential treatment targets in this specific patient population.

Recently, Paulus and colleagues proposed a novel hypothesis for the development HFpEF, which identifies a systemic proinflammatory state leading to microvascular endothelial inflammation induced by comorbidities (e.g. obesity, diabetes mellitus, hypertension, chronic obstructive pulmonary disease), as the cause of myocardial structural and functional alterations.[3] This model of a microvascular inflammatory state driving myocardial remodeling is further supported by the involvement of other cardiac chambers than the left ventricle in the progression of HFpEF.[3] There is growing evidence that outcome in patients with HFpEF strongly relies on pulmonary pressures and right ventricular function.[4–6] Using non-invasive echocardiographic surrogate measurements, Lam et al. previously demonstrated a significant prevalence of pulmonary hypertension in patients with HFpEF and a link between systolic pulmonary artery pressure (sPAP) and mortality.[4] However, in a recent study we could demonstrate a clear interdependence between the extent of LV extracellular matrix as quantified by cardiac magnetic resonance (CMR) imaging and parameters of RV afterload and function.[7]

Therefore, we aimed to comprehensively assess the impact of left- and right-ventricular function and hemodynamics on long-term mortality using invasive hemodynamic assessment, echocardiography and CMR imaging as well as histologic data obtained by myocardial biopsy.

## Materials and Methods

### Study Population

We prospectively included 142 consecutive patients diagnosed with HFpEF referred to the Department of Cardiology of the Medical University of Vienna between December 2010 and July 2013 into our observational, non-interventional registry. The Medical University of Vienna is a tertiary care center with a high-volume cardiac catheterization unit. The study protocol complies with the Declaration of Helsinki and was approved by the Ethics Committee of the Medical University of Vienna (EK #796/2010). Written informed consent was collected before study enrollment in all patients.

### Clinical Definitions

Diagnosis of HFpEF was defined according to the current consensus statement of the European Society of Cardiology [8] and the ACCF/AHA task force.[9] For the diagnosis of HFpEF all of the following diagnostic criteria had to be fulfilled: 1. clinical symptoms of heart failure (NYHA stage ≥ II) 2. an echocardiographic left ventricular ejection fraction ≥50% and a left ventricular end-diastolic volume index ≤ 97ml/m^2^ 3. evidence of abnormal left ventricular relaxation, filling or diastolic stiffness as previously described [7]. Pulsed-wave Doppler and Tissue Doppler Imaging were performed to obtain the ratio of early transmitral blood velocity (E) to early diastolic mitral annular velocity (e’). HFpEF was considered likely in patients with an E/eʼ ratio >15 and unlikely in patients with an E/e’ ≤ 8. In intermediate cases with 15 > E/eʼ ≥ 8, serum NT-proBNP levels were determined and if NT-proBNP levels exceeded 220pg/ml, HFpEF was considered. If HFpEF was expected after transthoracic echocardiography and N-terminal pro–brain natriuretic peptide assessment, right heart catheterization was performed. The diagnosis of HFpEF was established if the pulmonary artery wedge pressure (PAWP) was > 12mmHg.[10]

The presence of cardiovascular risk factors such as hypertension, diabetes mellitus, current smoking status, and lipid disorders, was recorded according to the respective guidelines. Furthermore, antecubital venous blood samples were drawn and analyzed directly without freezing according to local laboratory standard procedure.

Exclusion criteria were significant valvular or congenital heart disease, a significant coronary artery disease requiring percutaneous coronary intervention or aorto-coronary bypass surgery, and severe congenital abnormalities of the lungs, thorax or diaphragm as previously described.[7]

### Study Endpoints and Follow-up

Patients were prospectively surveyed in six-months-intervals by outpatient visits or telephone calls in cases of immobility. The primary study endpoint was a combined measure consisting of hospitalization for heart failure and/or death for cardiac reason.

### Imaging Modalities

All patients underwent a conventional transthoracic echocardiography (Vivid 5 and 7, General Electric Inc.) according to the guidelines of the American Society of Echocardiography.[11]

Two independent observers blinded to clinical data assessed RV function semi-quantitatively. An additional board-certified senior physician was consulted in case of disagreement. Right ventricular function was categorized semi-quantitatively into normal, mildly reduced, moderately reduced and severely reduced. Significantly impaired right ventricular function was defined as ≥ moderately reduced right ventricular function. End-diastolic and end-systolic volumes were used to calculate the ejection fraction using the Simpson’s biplane method on the apical four- and two-chamber views.[11]

Furthermore, all patients without contraindications underwent a CMR study on a 1.5-T scanner (Avanto, Siemens Medical Solutions, Erlangen, Germany). Studies consisted of functional and late gadolinium enhancement imaging, according to standard protocols.[12] Post-contrast T1 mapping was done 15 minutes after injection of 0.1mmol/kg gadolinium-DTPA [Gadovist 1.0 macrocyclic; Bayer Vital GmbH, Leverkusen, Germany]. A multiple breath-hold ECG-triggered segmented inversion recovery spoiled gradient echo sequence (fast low-angle shot) was used to acquire a stack of 8 images in the middle short-axis slice over a range of increasing inversion times from 115 to 900 ms as previously published [7]. Images were then transferred to an external computer for off-line T1 time analysis (cmr42; Circle Cardiovascular Imaging, Calgary, Canada).

Invasive hemodynamic assessment was performed in all study participants for a definite diagnosis of HFpEF. Hemodynamic measurements were performed using a 7F Swan-Ganz catheter (Edwards Lifesciences GmbH, Austria) via a jugular or femoral access. Pressures were documented as average of eight measurements over eight consecutive heart cycles using CathCorLX (Siemens AG, Berlin and Munich, Germany). In addition to PAWP, the systolic (sPAP), diastolic (dPAP) and mean (mPAP) PA pressures were documented. Cardiac output (CO) was measured by thermodilution. Furthermore, the transpulmonary gradient (TPG) and diastolic pulmonary vascular pressure gradient (DPG) were calculated as previously described [13]. TPG was computed by subtracting PAWP from mPAP; DPG was calculated as the difference between dPAP and PAWP during a pull-back; pulmonary vascular resistance (PVR) was calculated by dividing TPG by CO; pulmonary pulse pressure (PPP) was calculated as the difference between sPAP and dPAP; pulmonary arterial compliance (PAC) as the ratio of stroke volume (CO/heart rate) to PPP. Besides, coronary angiography was performed in all patients in order to exclude coronary heart disease. Additionally, 16 patients agreed to an endomyocardial biopsy taken from the left ventricle utilizing a Bipal biopsy forceps (Cordis Corporation a Johnson & Johnson Company, New Jersey, USA).

### Histochemistry and TissueFAXS Analysis

Myocardial biopsy samples were immediately fixed in 7.5% buffered formalin for at least 24 hours. Afterwards samples were dehydrated with an ascending ethanol row, embedded in paraffin and cut at 3μm using the Leica RM 2255 Microtome (Charleston, USA). The formaldehyde-fixed 3μm paraffin sections were stained with a modified trichrome stain according to a standardized protocol for further histochemical analysis.[14] Stainings were recorded by using a Zeiss Observer Z1 microscope (Carl Zeiss Microsocopy GmbH, Jena, Germany) and the TissueFAXS software (Version 3.5.5, TissueGnostics, Vienna, Austria) and then automatically analyzed using the HistoQuest software (TissueGnostics). Results were given as percentage extracellular matrix per mm^2^ of total heart area.

### Statistical Analysis

Continuous data were presented as median and interquartile range and discrete data were presented as counts and percentages. Cox proportional hazard regression analysis was applied to assess the effect of the respective variables on event-free survival. A multivariate model was adjusted for established cardiovascular risk factors in order to account for potential confounding. Adjusted hazard ratios were obtained by including the clinically established risk factors that were significant in our univariable analysis i.e. diabetes, COPD, and NT-ProBNP in our multivariate analysis. Estimated GFR was calculated using the Cockcroft-Gault formula. The correlations between the histological and imaging results were assessed using the Spearman correlation coefficient. Kaplan-Meier analysis (log-rank test) was applied to verify the time-dependent discriminative power of the respective variable. Receiver operating characteristic (ROC) analysis and Harrel’s C-statistic were used to assess the predictive value of the respective variables for the primary outcome. Continuous variables were divided into two groups using the median of the respective variable as cut-off value. Two-sided P-values <0.05 were used to indicate statistical significance. SPSS 18.0 (IBM SPSS, USA) and STATA 11 (StataCorp LP, USA) were used for all analyses.

## Results

### General Characteristics

The median age of HFpEF patients was 71 years (IQR 66–76), 70% of patients were female. We observed a high prevalence of previously established risk factors for HFpEF in our study population e.g. arterial hypertension (98%), diabetes (37%), chronic obstructive pulmonary disease (COPD, 36%) and a relatively high BMI with a median of 30 kg/m^2^ (IQR: 24–34). Sixty-two percent of HFpEF patients (n = 88) were in atrial fibrillation and NT-proBNP levels were elevated with a median of 1169 ng/L (IQR: 557–2024). Detailed baseline characteristics are displayed in *Table 1*. During a median follow-up time of 10 months (IQR: 5–19 months), 30% of patients (n = 43) reached the primary endpoint. Of these, 6 patients died due to cardiac causes. No patients were lost to follow-up.

**Table 1:** Baseline characteristics of patients with HFpEF (n = 142). Continuous variables are given as medians and inter-quartile ranges. Counts are given as numbers and percentages.

	HFpEF patients (n = 142)
Age, median years (IQR)	71 (66–76)
Female gender, n (%)	99 (70%)
BMI, kg/m^2^ (IQR)	30 (24–34)
Systemic hypertension, n (%)	139 (98%)
Current smokers, n (%)	55 (39%)
Atrial fibrillation, n (%)	88 (62%)
COPD, n (%)	51 (36%)
Diabetes mellitus, n (%)	52 (37%)
Hba1c, % (IQR)	5.9 (5.6–6.5)
NYHA class	
NYHA II, n (%)	42 (30%)
NYHA III, n (%)	87 (61%)
NYHA IV, n (%)	13 (9%)
6-min walking test, meter (IQR)	330 (240–418)
NT-proBNP, ng/L	1169 (557–2024)
Hyperlipidemia, n (%)	77 (54%)
Total cholesterol, mg/dl (IQR)	169 (144–199)
Triglycerides, mg/dl (IQR)	128 (91–161)
Serum creatinine, mg/dl (IQR)	1.1 (0.9–1.3)
GFR, mL/min/1.73 m^2^ (IQR)	68 (47–84)
C-reactive protein, mg/dl (IQR)	0.44 (0.2–1.0)
Medication	
Beta blocker, n (%)	88 (62%)
ACE inhibitor, n (%)	40 (28%)
Calcium channel blocker, n (%)	40 (28%)
Diuretics, n (%)	91 (64%)
Statin, n (%)	50 (35%)

BMI–body mass index, COPD–chronic obstructive pulmonary disease, NYHA–New York Heart Association, GFR–glomerular filtration rate, ACE–angiotensin-converting enzyme.

### Established Risk Factors and Outcome

We assessed the long-term effect of previously established risk factors for the development of HFpEF on hospitalization for heart failure and/or death for cardiac reason. Among these diabetes (HR 3.15, 95%CI 1.69–5.86; P<0.001) and COPD (HR 2.09, 95%CI 1.19–4.94; P = 0.02) were significantly associated with the primary outcome in the unadjusted analysis. Furthermore, increased NT-proBNP was a significant risk factor with an HR of 1.29 per 1 standard deviation (SD) increase (95%CI 1.07–1.56; P = 0.009). No significant association with outcome was detected for female sex (P = 0.29), age (P = 0.10), BMI (P = 0.22), and heart rate (P = 0.25).

### Non-invasive Imaging Measurements and Outcome

Among non-invasively measured parameters, the strongest univariable risk factors were reduced right ventricular function measured by echocardiography (HR 4.51; 95%CI 2.28–8.91; p<0.001) or CMR (right ventricular ejection fraction <35%: HR 8.11; 95% CI 2.37–27.75; p = 0.001) and sPAP using echocardiography (HR per 1-SD 1.76; 95%CI 1.34–2.32; p<0.001). These effects remained virtually unchanged after adjustment for potential confounders (*Table 2*). Furthermore, LA area measured with CMR was significantly associated with outcome with a HR of 1.41 (95%CI 1.03–1.95; p = 0.04) per 1-SD increase. However, this effect was not persistent in the multivariate Cox regression analysis. Detailed results of the Cox regression analysis are shown in *Table 2*.

**Table 2:** Cox proportional hazard models of non-invasive imaging measurements in patients with HFpEF (n = 142). Hazard ratios (HR) refer to a 1-SD increase in continuous variables. HRs are adjusted (adj.) for all variables in the clinical confounder model i.e. diabetes, COPD, and NT-ProBNP.

	Median (IQR)	Crude HR (95% CI)	P-value	Adj. HR (95% CI)	P-value
*Echocardiography*					
LA diameter, mm (IQR)	62 (58–66)	1.26 (0.97–1.65)	0.09	1.19 (0.88–1.61)	0.26
LV diameter, mm (IQR)	44 (40–47)	0.86 (0.65–1.15)	0.32	0.90 (0.66–1.23)	0.51
LVEF, % (IQR)	58 (65–64)	1.14 (0.79–1.63)	0.49	1.20 (0.81–1.76)	0.37
IVS thickness, mm (IQR)	12 (11–14)	0.88 (0.65–1.20)	0.43	0.79 (0.58–1.08)	0.14
RA diameter, mm (IQR)	62 (58–69)	1.24 (0.92–1.67)	0.17	1.22 (0.89–1.67)	0.23
RV diameter, mm (IQR)	37 (31–42)	1.46 (1.12–1.90)	**0.006**	1.41 (1.05–1.89)	**0.02**
Sign. impaired RV function, n (%)	17 (12)	4.51 (2.28–8.91)	**<0.001**	6.13 (2.85–13.16)	**<0.001**
sPAP (echo), mmHg (IQR)	56 (48–71)	1.76 (1.34–2.32)	**<0.001**	1.44 (1.07–1.95)	**0.017**
*Cardiac magnetic resonance*					
LA area, mm^2^ (IQR)	31 (26–35)	1.41 (1.03–1.95)	**0.04**	1.42 (1.03–1.97)	**0.04**
LVEDD, mm (IQR)	47 (44–50)	0.80 (0.55–1.19)	0.27	0.68 (0.44–1.06)	0.09
LVEDV, ml (IQR)	120 (102–139)	0.84 (0.55–1.27)	0.49	0.82 (0.54–1.25)	0.36
LVSV, ml (IQR)	76 (59–90)	0.74 (0.44–1.26)	0.27	0.77 (0.46–1.28)	0.31
LVEF, % (IQR)	62 (55–71)	1.10 (0.74–1.64)	0.64	1.14 (0.77–1.68)	0.53
IVS thickness, mm (IQR)	11 (10–13)	1.06 (0.75–1.50)	0.73	0.88 (0.62–1.25)	0.48
LV mass, g (IQR)	111 (92–138)	0.98 (0.64–1.49)	0.91	0.80 (0.52–1.23)	0.32
RA area, mm^2^ (IQR)	28 (24–35)	1.12 (0.76–1.64)	0.58	1.27 (0.81–2.00)	0.30
RVEDD, mm (IQR)	40 (36–44)	1.33 (0.92–1.91)	0.13	1.33 (0.88–2.01)	0.17
RVEDV, ml (IQR)	142 (116–172)	0.95 (0.63–1.43)	0.79	0.88 (0.60–1.29)	0.88
RVSV, ml (IQR)	75 (61–99)	1.01 (0.68–1.50)	0.94	0.97 (0.62–1.50)	0.88
RVEF, % (IQR)	51 (46–60)	0.73 (0.48–1.11)	0.14	0.75 (0.50–1.14)	0.18
T1 time myocardium, ms (IQR)	381 (349–433)	0.62 (0.38–0.99)	**0.05**	0.85 (0.52–1.38)	0.51
T1 time blood pool, ms (IQR)	272 (248–310)	1.22 (0.81–1.84)	0.35	1.48 (0.94–2.33)	0.09

LA–left atrium, LV–left ventricle, LVEF–left ventricular ejection fraction, IVS–interventricular septum, RA–right atrium, RV- right ventricle, sPAP–systolic pulmonary artery pressure, LVEDD–left ventricular end-diastolic diameter, LVEDV–left ventricular end-diastolic volume, LVSV–left ventricular systolic volume, RVEDD–right ventricular end-diastolic diameter, RVEDV–right ventricular end-diastolic volume, RVSV–right ventricular end-systolic volume, RVEF–right ventricular ejection fraction.

**Fig 1:**
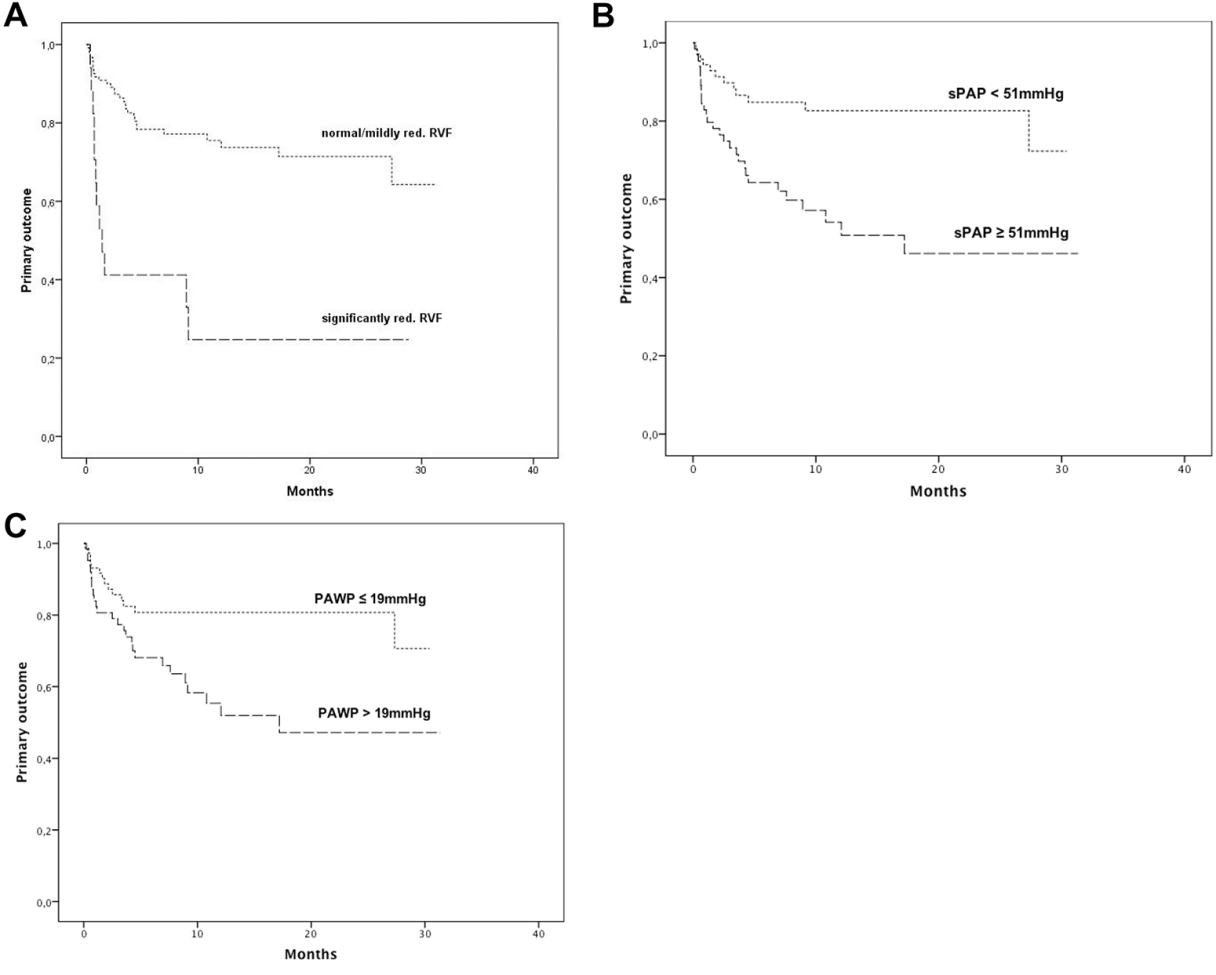
Kaplan-Meier estimates of primary endpoint (hospitalization for heart failure and/or death for cardiac reason) according to echocardiographic right ventricular function (normal and mildly reduced vs. significantly reduced. 3A; P<0.001, log-rank test), according to invasively measured systolic pulmonary arterial pressure (3B; cut-off = median; P = 0.001, log-rank test), and according to pulmonary artery wedge pressure (3C; cut-off = median; P = 0.006; log-rank test).

Kaplan Meier analysis demonstrated a significant increase of the primary endpoint, hospitalization for heart failure and/or death for cardiac reason, in patients with significantly reduced echocardiographic right ventricular function (*1A;* P<0.001, log-rank test). In detail, freedom from the primary outcome in patients with significantly reduced right ventricular function was present in 25% vs. 74% after 1 year (*Fig 1A*). The area under the ROC curve (AUC) in regard of the primary outcome was 0.63 for right ventricular function, with comparable results using Harrel’s C-statistic with a C for right ventricular function of 0.62.

### Invasive Hemodynamic Measurements and Outcome

Numerous invasive hemodynamic measurements were associated with the primary outcome, hospitalization for heart failure and/or death for cardiac reason, in the univariate Cox regression analysis and are displayed in detail in *Table 3*. In brief, in the univariate analysis invasively measured sPAP displayed a HR per 1-SD of 1.68 (95%CI 1.32–2.14; p<0.001), PAWP a HR of 1.56 (95%CI 1.18–2.06; p = 0.002), PVR a HR of 1.52 (95%CI 1.16–1.99; P = 0.002), and TPG a HR of 1.52 (95%CI 1.17–1.97; P = 0.002). These effects remained virtually unchanged after adjustment for potential confounders. Detailed results of the Cox regression analysis are shown in *Table 3*. Kaplan Meier analysis demonstrated a significant increase of the primary endpoint, in patients with increased sPAP (*Fig 1B*; cut-off = median; P = 0.001, log-rank test), and increased PAWP (*Fig 1C*; cut-off = median; P = 0.006; log-rank test). In detail, freedom from the primary outcome in patients with increased sPAP was present in 51% vs. 83% after 1 year (*Fig 1B*) and in patients with increased PAWP in 52% vs. 81% after 1 year (*Fig 1C*). The AUC was 0.75 for sPAP, and 0.68 for PAWP with corresponding C-statistics of 0.72 and 0.63, respectively.

**Table 3:** Cox proportional hazard models of invasive hemodynamic measurements in patients with HFpEF (n = 142). Hazard ratios (HR) refer to a 1-SD increase in continuous variables. HRs are adjusted (adj.) for all variables in the clinical confounder model i.e. diabetes, COPD, and NT-ProBNP.

	Median (IQR)	Crude HR (95% CI)	P-value	Adj. HR (95% CI)	P-value
SBP, mmHg (IQR)	136 (122–151)	0.77 (0.55–1.08)	0.13	0.77 (0.51–1.07)	0.11
Stroke volume, ml (IQR)	69 (56–85)	1.23 (0.90–1.68)	0.20	1.18 (0.86–1.62)	0.32
Cardiac output, l/min (IQR)	5.1 (4.3–6.2)	0.98 (0.70–1.37)	0.91	0.96 (0.69–1.35)	0.82
sPAP, mmHg (IQR)	51 (41–59)	1.68 (1.32–2.14)	**<0.001**	1.51 (1.15–1.98)	**0.003**
dPAP, mmHg (IQR)	22 (17–26)	1.74 (1.32–2.29)	**<0.001**	1.49 (1.10–2.03)	**0.01**
mPAP, mmHg (IQR)	33 (27–38)	1.71 (1.30–2.25)	**<0.001**	1.54 (1.13–2.10)	**0.006**
mRAP, mmHg (IQR)	12 (8–16)	1.68 (1.24–2.28)	**0.001**	1.60 (1.15–2.22)	**0.005**
PAWP, mmHg (IQR)	19 (16–23)	1.56 (1.18–2.06)	**0.002**	1.30 (0.98–1.71)	0.06
PPP, mmHg (IQR)	30 (21–37)	1.55 (1.22–1.97)	**<0.001**	1.41 (1.09–1.82)	**0.009**
PVR, dynes.sec/cm^5^ (IQR)	190 (144–272)	1.52 (1.16–1.99)	**0.002**	1.43 (1.05–1.95)	**0.023**
PAC, ml/mmHg (IQR)	2.4 (1.9–3.1)	0.60 (0.37–0.97)	**0.04**	0.66 (0.41–1.08)	0.10
TPG, mmHg (IQR)	13 (9–17)	1.52 (1.17–1.97)	**0.002**	1.47 (1.09–1.98)	**0.012**
AvDO_2_, ml O_2_/ 100ml (IQR)	4.9 (4.3–5.6)	1.24 (0.94–1.63)	0.13	0.97 (0.72–1.30)	0.83

SBP–systolic blood pressure, sPAP–systolic pulmonary artery pressure, dPAP–diastolic PAP, mPAP–mean PAP, mRAP–mean right atrial pressure, PAWP–pulmonary artery wedge pressure, PPP- pulmonary pulse pressure, PVR–pulmonary vascular resistance, PAC–pulmonary arterial compliance, TPG–transpulmonary gradient, SaO_2_ –arterial O_2_ saturation, SvO_2_ –central venous O_2_ saturation, AvDO_2_ –arterio-venous O_2_ difference.

### Fibrosis, LV Diameter and Stroke Volume

**Fig 2:**
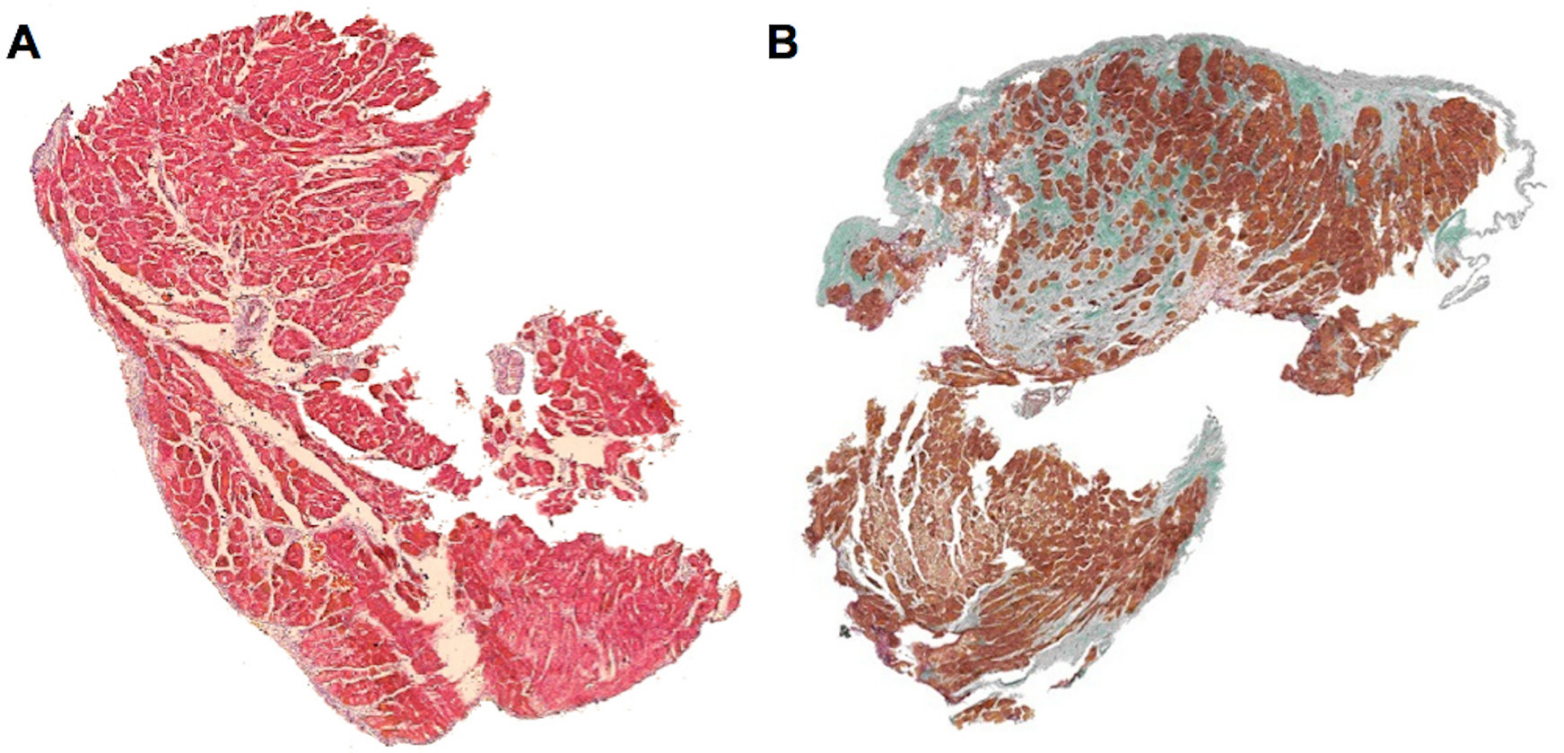
Left ventricular myocardial biopsies from HFpEF patients. Panel A shows a representative myocardial biopsy from a patient with a normal stroke volume (70ml) and a small extent of extracellular matrix (12%/mm^2^). In contrast, panel B shows a representative myocardial biopsy from a patient with a lower stroke volume (51 ml) and a significant amount of extracellular matrix (57%/mm^2^). Trichrome stain. Extracellular matrix stains blue-purple or green.

**Fig 3:**
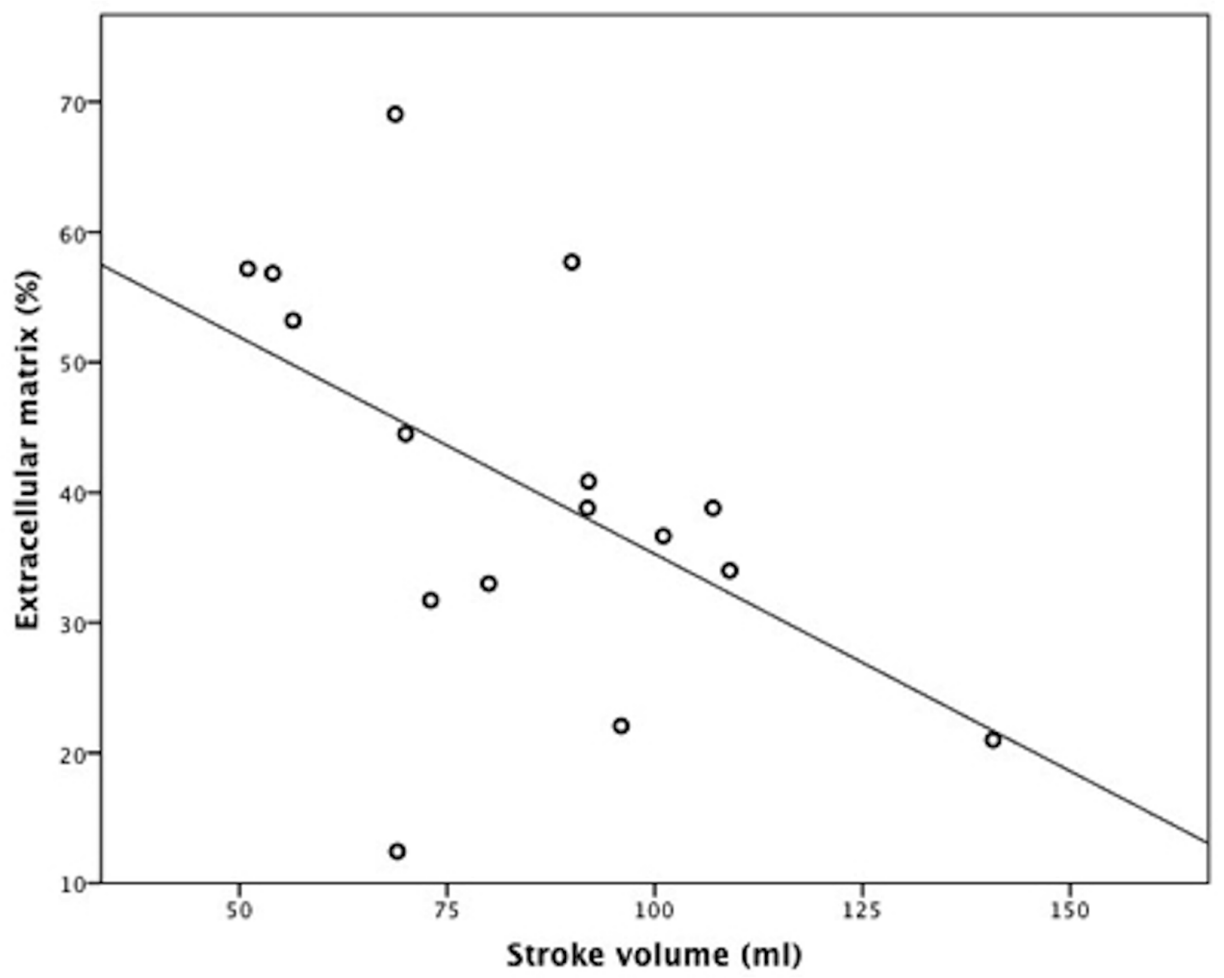
Correlation between the extent of left ventricular extracellular matrix and invasively measured stroke volume (r = -0.53; p = 0.04). Four patients with myocardial biopsy were not eligible for CMR study due to pacemakers.

We detected significant correlations between left ventricular extracellular matrix and invasively measured stroke volume (r = -0.53; p = 0.04; *Figs 2 and 3*). Interestingly, we could not detect any significant correlations between left ventricular extracellular matrix and PAWP (r = -0.02; p = 0.99) or sPAP (r = 0.16; p = 0.52). Moreover, we did not observe a correlation between extracellular matrix and LV mass (r = 0.05, P = 0.89) by MRI or the thickness of the interventricular septum measured by MRI (0.36, P = 0.25), or echocardiography (r = 0.18, P = 0.49). Additionally, we did not detect substantial differences in baseline characteristics between patients with and without myocardial biopsy (data not shown).

## Discussion

The current study demonstrates the importance of right ventricular performance on outcome in patients with HFpEF. We detected a strong association between right ventricular function as well as pulmonary pressures and hospitalization for heart failure and/or death for cardiac reason. These associations were independent of the utilized method (i.e. echocardiography, CMR and invasive hemodynamic measurement) and even more pronounced after adjustment for potential confounders. There was also an association between left ventricular filling pressures and outcome. Moreover, we detected a significant correlation between the extent of extracellular matrix obtained by left-sided myocardial biopsy and stroke volume.

### HFpEF and Right Ventricular Performance

The fact that right ventricular performance is crucial of prognosis in heart failure has been previously demonstrated by Ghio et al., who followed 377 heart failure patients with reduced ejection fraction and identified both sPAP and RV systolic function as independent prognostic predictors.[15] Furthermore, Burke et al. demonstrated that beyond remodeling of the RV decreased LV compliance, measured as reduced LV end-diastolic volume at an idealized LV end-diastolic pressure of 20 mm Hg, was the pathophysiologic marker most predictive of worse outcomes in HFpEF.[16] More recently, Melenovsky et al. showed that RV dysfunction was the strongest predictor of death in HFpEF.[5] Mohammed et al. acquired similar results using semiquantitiative echocardiographic assessment of right ventricular function and the tricuspid annular plane systolic excursion.[6] In contrast to the aforementioned studies, our RV functional assessment is not solely based on echocardiography but further confirmed by CMR and most importantly complemented by the availability of invasive hemodynamic measurements. While Lam and co-workers [4] have clearly demonstrated that echo Doppler estimates of sPAP predict outcome in HFpEF, the anatomical sites involved in pressure elevations have not been studied. In fact, the differentiation between pre- and postcapillary PH can only be obtained by RHC. Besides elevations in PAWP, we found that the presence of pulmonary vascular disease as reflected by increased PVR and TPG predicts outcome in HFpEF.

Moreover, recent autopsy studies demonstrated significant cardiac hypertrophy, coronary microvascular rarefaction and myocardial fibrosis in patients with HFpEF compared to controls.[17] We further extend these previous results by adding information on LV myocardial structure obtained by myocardial biopsy. We found a strong inverse correlation between the extent of the left ventricular extracellular matrix and the invasively measured stroke volume. In fact, structural changes in both heart chambers seem to independently predict outcome: left ventricular remodeling processes with stiffening, filling impairment and consecutive rise in left ventricular filling pressures on one hand and right ventricular systolic dysfunction due to an increased afterload with variable degrees of pulmonary vascular remodeling on the other hand are major determinants of the clinical course [13]. Thus, estimates of sPAP by transthoracic echocardiography together with a visual assessment of the RV may be appropriate to predict prognosis in HFpEF patients. Interestingly, we did not observe a significant correlation between LV filling pressures or LV mass and myocardial fibrosis obtained by myocardial biopsy. This may suggest that myocardial fibrosis although a significant driving force in the development of HFpEF might not be the sole trigger for an increase in LV filling pressures in the pathogenesis of HFpEF. Recent data by Zile et al. suggests that myocardial stiffness in patients with HFpEF not only depends on the extent of extracellular matrix, but also on myocardial titin homeostasis, which might explain our findings [18]. However, our results need to be interpreted with caution since our number of LV biopsies is rather small and our histology-based analysis should be more considered as hypothesis-generating and foster future investigations.

Among known clinical risk factors for the development of HFpEF, only atrial fibrillation and COPD were associated with adverse outcome. Zakeri et al. propose that atrial fibrillation serves as a marker of disease severity in patients with HFpEF and demonstrated a significant association between atrial fibrillation and exercise capacity [19]. Moreover, atrial fibrillation has been demonstrated to be a potent risk factor for adverse outcome in a contemporary cohort of HFpEF patients.[20, 21] Our results confirm previous findings by revealing a significant association between atrial fibrillation and hospitalization for heart failure and/or death for cardiac reason. In congruence, left atrial diameter as determined by echocardiography was identified as a useful imaging parameter of significant predictive value. We further found an association between BMI and poor outcome, however only borderline statistical significance was reached. This finding is compatible with previous observations made by Kapoor et al, who described a U-shaped relationship between obesity and survival in HFpEF patients.[22] Furthermore, we were able to confirm previous results indicating a significant association between COPD and outcome in patients with HFpEF in our well-characterized study population.[23, 24]

### Clinical Implications

Our findings suggest that outcome in patients with HFpEF strongly depends on both the systemic as well as the pulmonary vascular system. Moreover, accumulation of extracellular matrix in the LV wall with consecutive stiffening and reduction in stroke volume seems to be the central pathobiological change in HFpEF. Progression of the disease is accompanied by progressive remodeling of the pulmonary vasculature, increased RV afterload, and finally RV failure. Although therapeutic interventions targeting the pulmonary vascular system might be promising, a more profound understanding of the mechanisms underlying LV and pulmonary vascular disease in HFpEF and time course of the remodeling processes will be necessary to develop and apply disease state-specific drugs. Interestingly, recent data by Andersen and Hwang et al. determined that pulmonary vascular dysfunction in early stage HFPEF is partly reversible and responsive to β-adrenergic stimulation [25]. This might suggest that the pulmonary vasculature in HFpEF is not fully vasodilated at rest, and that interventions to enhance β-receptor activation in the pulmonary vasculature may be a therapeutic target. Additionally, RV-PA coupling was altered together with RV systolic dysfunction, even in the absence of structural remodeling, providing further support for therapies targeting RV and pulmonary vascular function as novel approaches to improve outcomes in HFpEF [25].

### Study Limitations

One potential limitation of our study is that our data reflect the experience of a single tertiary care center. Therefore, a center-specific bias cannot be excluded, and all results and conclusions should be interpreted with caution. However, the major advantages of limiting data collection to a single center are inclusion of a homogenous patient population, adherence to a consistent clinical routine, as well as a consistent quality of imaging procedures and right heart catheterization. Furthermore, our patient population represents a more advanced spectrum of patients with HFpEF, which is supported by the high prevalence of atrial fibrillation (62% in our cohort versus 19% in the CHARM HFpEF cohort [26]), the large extent of myocardial fibrosis and relatively high pulmonary pressures. However, the inclusion of patients in more advanced stages of disease progression might ease the identification of disease specific pathophysiologic changes since they might appear more pronounced. Furthermore, the small number of events and the low number of deaths might limit the ability to discern whether the observed factors are truly independent. Another potential limitation is that myocardial biopsies were only available in sixteen patients. However, considering the invasive character and the potential complications of this procedure, the obtained number of histologic samples is still unique in prospectively enrolled patients complemented by invasive, echocardiographic and CMR measurements. We could not find a correlation between the extent of extracellular matrix and parameters reflecting LV filling pressures. In addition to structural changes in the LV, patients’ actual volume status is a crucial determinant of filling pressures. We did not systematically perform bioimpedance measurements to determine fluid status at right heart catheter, which could have complemented our histologic data on tissue composition and the understanding of LV hemodynamics. Further, we did not invasively measure LV compliance [27]. Therefore, the relation between extent of extracellular matrix and LV diastolic distensibilty remains unknown.

## Conclusions

The current study emphasizes the importance of right ventricular function and pulmonary pressures on outcome in patients with HFpEF providing pathophysiological insights into the hemodynamic changes in HFpEF. Our results were independent of the imaging method and complemented by histological data. Furthermore transthoracic echocardiography appears to be an easily available method to identify high-risk patients in HFpEF.
